# Outcome at Skeletal Maturity of Femoral Varus Osteotomy on Late Onset Legg-Calvé-Perthes Disease

**DOI:** 10.5704/MOJ.2511.014

**Published:** 2025-11

**Authors:** MA Ibrahim, M Phandu, MH Zahri, I Munajat, EF Mohd, AR Sulaiman

**Affiliations:** 1 Department of Orthopaedics, Hospital Tanjung Karang, Kuala Selangor, Malaysia; 2 Department of Orthopaedics and Traumatology, Universitas Pelita Harapan, Tangerang, Indonesia; 3 Department of Orthopaedics, Universiti Sains Malaysia, Kubang Kerian, Malaysia

**Keywords:** Legg-Calvé-Perthes disease, late onset, femoral varus osteotomy, containment, hinge abduction

## Abstract

**Introduction:**

Late-onset Legg-Calvé-Perthes disease, which is diagnosed in children older than 8 years old, is considered to have a poorer outcome. The outcome depends on the severity of the disease, the age at presentation, and the method of treatment. This study aims to evaluate the effectiveness of containment using femoral varus osteotomy (FVO) in late-onset Legg-Calvé-Perthes disease patients.

**Material and methods:**

We reviewed seven patients with Legg-Calvé-Perthes disease aged nine and older (four Herring B and three Herring C) who had containable hip included two cases of pseudo hinged abduction. All patients went through FVO at the subtrochanteric level, with the varus adjustment guided by an image intensifier to ensure hip containment in a neutral position, and the site was then fixed with a plate. The outcomes were assessed based on Stulberg classification of radiograph at skeletal maturity with a mean follow-up of 9.99 years.

**Results:**

All seven patients that went through FVO were between nine to eleven years old. Among the four Herring B patients, one had Stulberg I, one had Stulberg II, and two had Stulberg III. All three Herring C patients had Stulberg III.

**Conclusion:**

Femoral varus osteotomy produces a reasonable outcome in patients of nine years and older, including those with pseudo-hinge abduction.

## Introduction

Legg-Calvé-Perthes disease is uncommon disease with the incidence ranges from 0.4/100,000 to 29.0/100,000 children younger than 15 years of age^1^. It is an idiopathic avascular necrosis of the femoral head in the paediatric population, which is a self-limiting disease through ischemia, revascularisation, and remodelling^2,3^. The affected femoral head undergoes avascular necrosis for 6 to 12 months^2^. During the revascularisation phase, the dead osteoid bone is resorbed by osteoclast, making the femoral head relatively soft or “biologically plastic” and susceptible to microfracture and deformation because of weight bearing and muscular forces acting from the pelvic to the femur across the hip joint^3^.

The disease is usually diagnosed at 4 – 12 years old. The younger patient tends to have less lateral pillar involvement, has time for healing, and has a more spherical femoral head. Late-onset cases after the age of 8 are considered more aggressive and have poorer outcomes^4^. This is because they have more severe lateral pillar involvement, need more time for re-ossification, and have a higher risk of flattening the femoral head^5,6^. Treatment strategies for this group pose unique challenges and controversies for orthopaedic management.

The basic concept of treatment is that the femoral head has to be contained inside the acetabulum during the period of fragmentation to mould the femoral head smoothly and prevent it from deformation, limited range of motion, and early osteoarthritis^7,8^. Containment procedures can be done through abduction bracing, femoral varus osteotomy (FVO), or acetabuloplasty^2-4,8^. Osman *et al* found initial head deformity did not remodel for patients older than eight and progressed despite bracing or varus osteotomy but not after acetabular augmentation^9^. In our case series, we reviewed all the Legg-Calvé-Perthes patients aged 9 years and above who underwent FVO and followed-up until skeletal maturity.

However, patients with advanced stages of the disease may present with hinge abduction that prevents containment, thus limiting treatment options^10,11^. Therefore, we also reviewed cases with hinge abduction. This study aimed to determine the outcome of containment using FVO in patients with advanced age presentation. It is also meant to study associated factors like classification, and pseudo hinges at presentation to determine the outcome.

## Materials and Methods

This study was approved by the research ethical board at a tertiary centre. with code USM/JEPeM/20060335. A list of patients was obtained from the record office according to the ICD 10 classification of disease with the diagnosis of Legg-Calvé-Perthes disease in our centre from 2000 until 2023. Those patients who attain maturity and closed triradiate cartilage by 2023 were included in the study.

We included patients diagnosed with Legg-Calvé-Perthes disease at age nine or older who were proven to have containable hip on pelvis anteroposterior radiograph and underwent FVO for containment. The osteotomy was done at the subtrochanteric level, with the amount of varus done based on an image intensifier showing the intra-operative containment of the hip in a neutral position. The age of the patient at presentation, gender, presenting symptoms, and treatment received were recorded. The classification was made during the fragmentation phase of the disease based on AP radiograph using Herring lateral pillar classification, with group A having no loss of femoral height, group B measuring between 50-100 % of original height, and group C measuring less than 50% of original height^12^. The final follow-up radiographs were studied and classified according to the Stulberg classification when they reached skeletal maturity. Stulberg I no coxa magna, normal acetabulum and normal neck. Stulberg II either coxa magna, step acetabulum or shock neck. Stulberg III ovoid femoral head. Stulberg IV flat femoral head with coxa magna or steep acetabulum. Stulberg V coxa plana, collapsed and distorted, incongruent^13-15^. We used an anteroposterior pelvic radiograph for the final Stulberg assessment by unblinded two observers. All the radiographs were obtained from our Picture Archiving and Communication System (PACS).

## Results

**Fig. 1:**
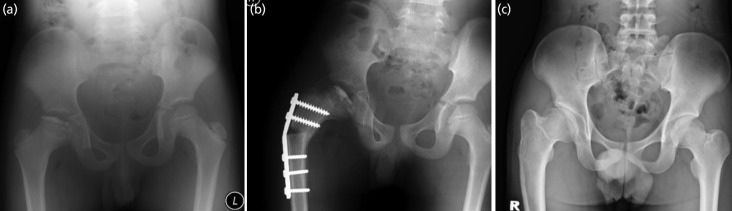
The series of radiographs belongs to patient no.1. (a) The presentation at the age of 9 showed a fragmentation phase with Herring C. (b) The immediate post-surgery showed good containment. (c) The hip at the age of 19 showed Stulberg III.

**Fig. 2:**
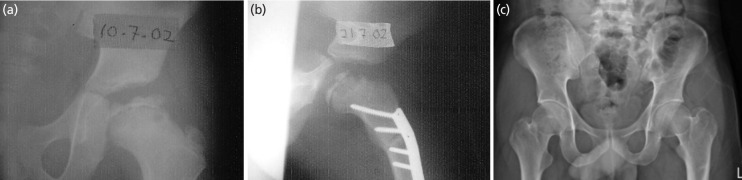
The series of radiographs belongs to patient no.2. (a) The presentation at the age of 9 showed fragmentation phase with Herring B. (b) The immediate post-surgery showed good containment. (c) The hip at the age of 28 years old showed Stulberg I.

**Fig. 3:**
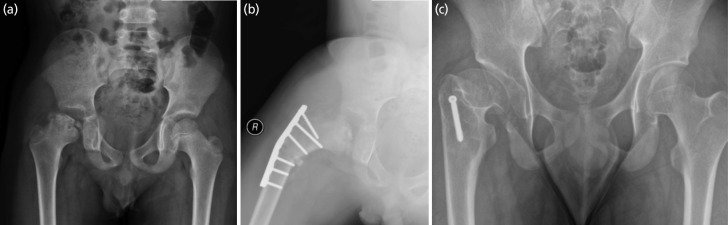
The series of radiographs belongs to patient no.3. (a) The presentation at the age of 9 showed fragmentation phase with Herring C. (b) The immediate post-surgery showed good containment. (c) The hip at the age of 16 years old showed Stulberg III. Overgrowth of the greater trochanter with the presence of a screw indicates failure of trochanter epiphysiodesis done at the age of 10 years old.

**Fig. 4:**
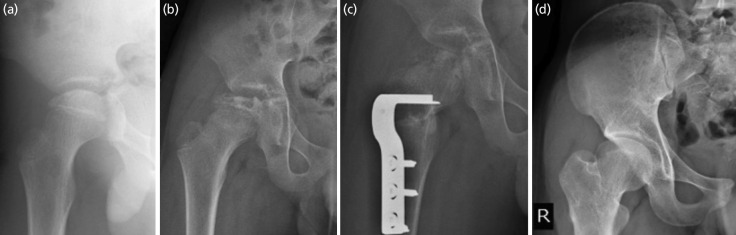
The series of radiographs belongs to patient no.4. (a) The presentation at the age of 10 showed ischemic phase. (b) Early fragmentation phase with Herring B at age of 11. (c) The immediate post-surgery showed good containment. (d) The hip at 21 years old showed good outcome with Stulberg II.

**Fig. 5:**
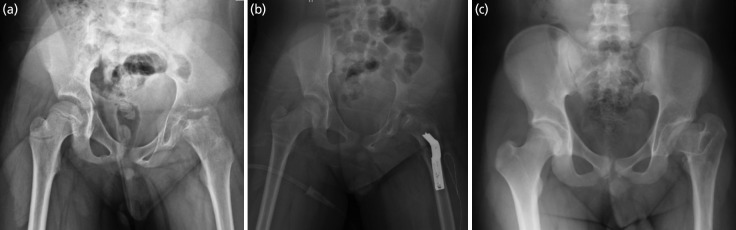
The series of radiographs belongs to patient no.5. (a) The presentation at the age of 10 showed fragmentation phase with Herring C. (b) The immediate post-surgery showed good containment. (c) The hip at the age of 17 years old showed Stulberg III.

**Fig. 6:**
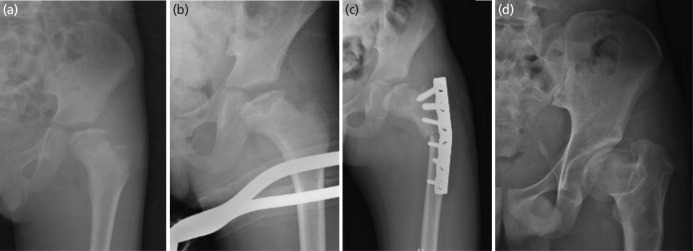
The series of radiographs belongs to patient no.6. (a) The presentation with Herring C and pseudo hinge abduction at age 11 years old. (b) The hip reduced with traction for 10 days. (c) The immediate post-surgery showed good containment. (d) The hip at maturity showed relatively greater trochanteric overgrowth, coxa magna, coxa breva and classified as Stulberg III.

**Fig. 7:**
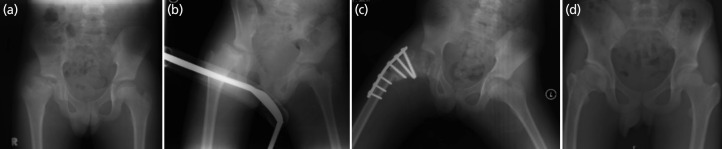
The series of radiographs belongs to patient no.7. (a) The presentation with Herring B and pseudo hinge abduction at the age of 11. (b) Failure of containment with traction. (c) The hip showed good containment after general anaesthesia, released of adductor muscles and FVO. (d) The hip at the age of 16 showed Stulberg III.

There were seven patients during 2000-2023 at our centre that met the criteria of the study. Six hips presented at the fragmentation phase and one hip at the ischemic phase. Four hips with Herring B and three with Herring C. All hips underwent a containment procedure at the fragmentation phase with FVO and fixation either with cannulated paediatric osteotomy system (CAPOS) plate or dynamic compression (DCP) plate. All three Herring C patients had Stulberg III. Among the four Herring B patients, one had Stulberg I, one had Stulberg II, and two had Stulberg III (Table I, Fig. 1-7).

**Table I:** List of patients with the clinical feature at initial presentation, method of treatment and outcome.

Patient no.	Age (years) / gender	Pseudo-hinge abduction	Stage I/F/R	Herring classification at presentation	Procedure	Stulberg classification	Age at last follow-up (duration of follow-up)
1.	9/m	No	F	H-B	FVO - DCP	III	19 (10 years)
2.	9/m	No	F	H-B	FVO - DCP	I	28 (21 years)
3.	9/m	No	F	H-C	FVO - DCP	III	17 (8.7 years)
4. *	10/m	No	F	H-B	FVO - CAPOS	II	21 (9.8 years)
5.	10/m	No	F	H-C	FVO - CAPOS	III	17 (7 years)
6.	11/m	Yes	F	H-C	FVO - DCP	III	19 (7.8years)
7.	11/m	Yes	F	H-B	FVO - DCP	III	16 (5.6years)

Abbreviations – F: fragmentation, I: ischemic, R: remodelling, H: Herring, CAPOS-cannulated paediatric osteotomy system, DCP-dynamic compression plate, * This patient presented at 10 years old with an ischemic phase and followed up until developed Herring B at age of 11 with fragmentation phase when the FVO was done.

Patients with early presentation (ischemic phase), even at age of 10, had Stulberg II at maturity after going through 8 weekly follow-up treatment and FVO at the age of 11 with early fragmentation phase (Fig. 4).

Patient no.6, with Herring C (Fig. 6) and limited abduction at 11 years old, could attain containment with traction. While patient no.7, with with Herring B (Fig. 7) and limited abduction at 11 years old, could not attain containment by traction and was able to attain containment with general anaesthesia and adductor tenotomy. Both patients went through FVO and achieved Stulberg III.

## Discussion

Outcomes of surgical treatment were significantly better than those of non-operative treatment in children over the age of 8 years at the onset of the disease^4,16^. However, there are still different opinions on the surgical treatment results. Osman *et al* said the containment procedure, either by FVO or brace, after 8 years did not guarantee a significant head improvement compared to the no-treatment group^9^. While Herring *et al*^4^ stated that children in lateral pillar group C have an unfavourable prognosis with surgical treatment regardless of age. However, Lim *et al* have shown a good to moderate outcome in 11 out of 12 patients with lateral pillar C treated with a combination of shelf acetabuloplasty and FVO containment procedures in above 8-year-old patients^17^. While Javid *et al* found that a combination of FVO and salter innominate in over 8-year-old patients with Herring groups B and C produced a good to fair outcome^18^. This is consistent with our finding where all patients had Stulberg I to III, which is considered a good to moderate outcome as proposed by Herring *et al*^4^. This finding indicates that containment would still be important for patients older than eight years as long as the disease is still in the fragmentation phase.

We performed femoral osteotomy as a containment procedure to ensure the femoral head remained in the acetabulum through the period of revascularisation^5^. Another choice of surgical containment, Salter osteotomy, was known to maintain neck shaft angle, length, and abduction movement^19,20^. Shelf acetabuloplasty with FVO was found to have good clinical outcomes at the age of 8 years or older^17^. We preferred to do only FVO due to its simpler technique. Furthermore, femoral varus osteotomy has no significant difference in outcome compared to innominate osteotomy^4^. The slight shortening resulting from FVO may give further hip containment due to pelvic tilt^21^. Limb length discrepancy has not been causing significant problems because of the remodelling of deformity and overlengthening that usually occurs within three years after surgery^22^.

Among this limited number of late-onset Legg-Calvé-Perthes disease patients, we found that Herring C patients resulted in Stulberg III while less severe patients had better outcomes. We saw a good result in patient no.4, who presented in the ischemic phase at the age of 10 and went through FVO at age 11, when he progressed to the early fragmentation phase with Herring B. Meanwhile, patient no.2 with Herring B had better results, Stulberg I. This indicates different patients may behave differently. Furthermore, this study was based on chronological age, while patient no.2 could have a younger skeletal age, which we could not prove in this retrospective study. Thus, judgment should not be made only based on chronological age.

Hinge abduction in Legg-Calvé-Perthes disease was defined as the limitation of hip abduction due to impingement of the outer part of the deformed femoral head onto the lateral lip of the acetabulum in abduction^6,10^. The reported incidence of hinge abduction in Legg-Calvé-Perthes disease ranges from 4% to 70%^23^. In the presence of hinge abduction and an uncontainable hip, a containment procedure like FVO or innominate osteotomy is inappropriate.

Limitation of hip abduction can also be due to synovitis, pain, or spasm of the adductor muscles in early Legg-Calvé-Perthes disease, which is still reversible with traction or general anaesthesia; this is known as pseudo-hinge^14,24^. Therefore, assessment under general anaesthesia and muscle relaxants before confirming a true hinge abduction is needed^24^. There is also a suggestion for traction for several weeks, followed by a cast or various surgical procedures such as femoral or pelvic osteotomy^23^. Patients no.6 and no.7, with pseudo-hinge abduction, were put on traction for 10 days, followed by femoral head containment under general anaesthesia and confirmed with an arthrogram. Both patients went through FVO and obtained Stulberg III at maturity. With this experience, we suggest treating the patients with pseudo hinge abduction by traction and putting the patient under general anaesthesia with options for either FVO for a containable hip or salvage procedure for an un-containable one.

Despite the small sample size, this case series' strength was that it reported uncommon late presentations that were containable with FVO and followed-up until skeletal maturity. One limitation of this study was that the assessment relied solely on radiographs due to its retrospective nature.

## Conclusion

Our finding suggests that FVO containment produces reasonable outcomes in patients between 9 to 11 years old. It also could still be an option for patients with pseudo-hinge abduction.
